# Identification investigations: a collaborative approach to the resolution of long-term unidentified persons cases at the NYC Office of Chief Medical Examiner

**DOI:** 10.1093/fsr/owae039

**Published:** 2024-07-20

**Authors:** Angela Soler, Justin Z Goldstein, Aden Naka, Stephanie Santiago

**Affiliations:** Forensic Anthropology Unit and Identifications Unit, New York City Office of Chief Medical Examiner, New York, NY, USA; Forensic Anthropology Unit and Identifications Unit, New York City Office of Chief Medical Examiner, New York, NY, USA; Forensic Anthropology Unit and Identifications Unit, New York City Office of Chief Medical Examiner, New York, NY, USA; Forensic Anthropology Unit and Identifications Unit, New York City Office of Chief Medical Examiner, New York, NY, USA

**Keywords:** forensic sciences, forensic anthropology, identification, unidentified persons, long-term unidentified

## Abstract

The New York City Office of Chief Medical Examiner (NYC OCME) investigates approximately 9 000–10 000 deaths every year, each of which necessitates a formal identification. Although standard identification protocols resolve the majority of these cases, there are still a substantial number of long-term unidentified persons cases that require a targeted investigation. This process involves not only the comprehensive review of all available postmortem data (e.g. scene findings, personal effects, autopsy findings, toxicology results, forensic anthropology reports, dental findings, fingerprints, forensic biology), but also the collection of antemortem data through focused informant interviews, analyzing casefiles and/or archival records, reviewing public missing person postings (e.g. National Missing and Unidentified Persons System (NamUs)), and collaborating with law enforcement and other agencies. This holistic approach to identification investigations is systematic yet flexible, allowing for the needs of each unidentified person and/or missing person case to be thoroughly assessed and efficiently addressed. These efforts have proven successful at NYC OCME, resulting in over 80 long-term unidentified persons identifications confirmed in the last 7 years, dating as far back as 1969. This paper provides a detailed breakdown of the NYC OCME framework for long-term unidentified persons investigations, citing multiple case studies to underscore how investigators utilize multiple lines of evidence to generate potential leads. Although each jurisdiction faces a unique set of demands and limitations, sharing these investigative strategies and perspectives may benefit practitioners contending with long-term unidentified persons cases and their inherent complexities.

**Key points:**

- The NYC OCME oversees a large caseload of unidentified persons yet achieves a high rate of case resolution by applying a systematic and holistic investigation that involves a comprehensive review of postmortem data and the collection of antemortem data specific to a forensic identification.
- Analyzing multiple lines of postmortem biological and contextual evidence allows practitioners to hypothesize possible socioeconomic, cultural, and religious social identities that can be used to generate identification leads and inform comparisons to missing persons.
- Gathering as much detailed, accurate, and comprehensive antemortem data for a missing person is equally important when performing an identification comparison to a long-term unidentified persons (UP) and can better inform archival morgue records searches.

## Introduction

As of 2024, there are over 14 000 unidentified persons cases within the USA that are currently uploaded to the National Missing and Unidentified Persons System (NamUs). Due to the large variation between jurisdictions regarding compliance and timeframes for posting cases to NamUs, this number underestimates the true number of unidentified individuals. Some of these individuals were recovered decades ago and the majority can be considered “true unknowns” in that there is no tentative information regarding identity. Such cases represent some of the most complex identification investigations, requiring practitioners to examine biological and contextual evidence and, most importantly, apply a well-informed interpretation of multidisciplinary findings. Analyzing these lines of evidence allows practitioners to hypothesize possible socioeconomic, cultural, religious, and social identities that can be used to generate identification leads and inform comparison to missing persons reports.

In New York City (NYC), the challenges inherent in identification investigations are exacerbated given the city’s large, globalized population (approximately 8.5 million) and massive tourist population (approximately 60 million per year), not to mention commuters from Upstate New York, Long Island, Connecticut, New Jersey, and Pennsylvania. In addition, NYC is home to substantial unhoused, undocumented, and transient populations. The intersecting and ever-changing nature of the city’s population makes it particularly difficult for identification investigations—an unidentified individual could have come from anywhere in the country, let alone across the globe. It is with this in mind that the NYC Office of Chief Medical Examiner (NYC OCME) investigates 9 000–10 000 deaths per year, each requiring a formal identification.

Due to the nature of casework in NYC, the NYC OCME has instituted a holistic approach to identification investigations, as experience has demonstrated its importance in both daily and long-term unidentified persons (UP) investigations. First, identification leads must be generated before a comparison and confirmed identification are possible. Most identification leads at the NYC OCME are generated through scene documentation, fingerprint searches, NamUs, and thorough analysis of postmortem physical and contextual information. Few identification leads at the NYC OCME are generated through blind Combined DNA Index System (CODIS) rank matches, with an average of approximately 5 blind CODIS rank matches each year (<1% annually). In cases where fingerprints are not available or do not yield results, a broad analysis of the postmortem physical and contextual information is the most efficient, and sometimes only, way to elicit an identification lead. Furthermore, even when a scientifically based identification lead is generated (e.g. fingerprint match or CODIS offender ranking), the information provided is investigated to verify the identification and locate next of kin. Since it is not possible to predict which antemortem or postmortem information will be key in generating an identification lead, the data collection process must be detailed and all encompassing. The NYC OCME has developed a model to conduct effective identification investigations and applies those methods when investigating long-term unidentified person (UP) cases. In outlining how long-term unidentified cases are approached at the NYC OCME and utilizing multiple case studies, this paper demonstrates how forensic investigators can apply a comprehensive analysis of postmortem evidence and collection of antemortem missing persons information to generate identification leads suitable for comparison purposes. Although the application of this model will differ based on the needs of each jurisdiction, this paper provides additional perspectives for practitioners to implement in their own casework.

## The NYC OCME identification procedures

Before discussing long-term UP cases, it is essential to review the current standard investigative procedures that are conducted prior to characterizing someone as a long-term UP. A formal identification is required for each case where jurisdiction is accepted and a death investigation is performed by the NYC OCME. Identification modalities include visual, contextual, fingerprint, medical or dental comparative radiography, and DNA. Interdepartmental and interagency collaboration is key, especially in more complex identification cases, and may include information collected from Medicolegal Investigations, Mortuary, Forensic Pathology, Identifications and Outreach, Forensic Anthropology, Radiology, Forensic Odontology, and Forensic Biology, as well as the Federal Bureau of Investigation (FBI) and local law enforcement.

The identification investigation begins at the scene with the Medicolegal Investigators (MLIs). MLIs interview individuals at the scene, document the decedent *in situ*, and record contextual information, such as the location and position of the body, body habitus, personal effects, medical and identification documents, and any information that may direct the investigation. Once the decedent is transported to the morgue, staff from the Mortuary and Forensic Pathology departments will collect postmortem data that include visual identification photos, fingerprints, DNA samples, and documentation of the decedent’s personal effects, physical features, and medical findings. Fingerprints are electronically acquired using a digital fingerprint scanner, or through advanced techniques such as inking or casting by NYC OCME staff. Utilization of multiple capture techniques is encouraged, as multiple images of the same fingerprints can be submitted for evaluation. Obtaining fingerprints is a critical step in the identification investigation and should always be attempted, including in cases where advanced decomposition or mummification has occurred and more complex acquisition techniques are required. Even if only one finger yields a print of sufficient quality, that print can still match an existing record and help confirm an identification. Different fingerprint acquisition techniques are utilized in order of least invasive (digital scanning) to most invasive (boiling, chemical rehydration). Once acquired and formatted, fingerprints are submitted by the NYC OCME for automated search through NY state and federal fingerprint databases, regardless of identification status. Depending on the quality of the fingerprints and the initial results, the OCME may also forward the fingerprints for manual latent print searches or to additional fingerprint databases.

Within the Identification Unit, Identification Investigators utilize the available postmortem data to pursue leads and confirm decedent identification. This includes vetting fingerprint response data, collecting detailed contextual information from family or other informants, coordinating visual identification, and/or collecting a DNA family reference sample, if needed. This also includes searching various governmental and non-governmental databases for records, requesting dental or medical radiographs, and referring information to external agencies to locate identifying information and potential next of kin.

If an individual remains unidentified after initial efforts have concluded, the case is forwarded to the Forensic Anthropology Unit (FAU) for entry into NamUs. FAU staff upload, manage, and track all the NYC UP cases in NamUs. These uploads include both public information about the individual (e.g. unique characteristics, tattoos, personal effects, any tentative names/aliases), as well as private information available only to NamUs practitioner users (e.g. medical data, dental charting and radiographs, DNA testing, and fingerprint submissions). The FAU will also conduct a review of radiographs for possible identifying features, such as prior trauma and/or surgery, and will obtain skeletal samples to provide a scientific age estimation. In instances where the individual is decomposed, burned, skeletonized, or fragmentary, the FAU may also assess additional components of the biological profile such as sex, population affinity, and stature.

If no additional identification leads are generated, the case will be prepared for the monthly Identification Review Committee (IRC). The IRC is a multidisciplinary team of identification specialists from various OCME units who meet monthly to review complex identification cases, such as “true unknowns”, and individuals with tentative identities that could not be verified using the standard modalities [1]. The IRC recommends next steps that may include a confirmed identification, to pursue additional investigative avenues or DNA testing, or to release to Hart Island (the NYC public cemetery) with an unverified name or as a true unknown. Prior to release to Hart Island, the case is reviewed to ensure that all available and actionable leads have been exhausted and all postmortem data collection has been completed. This includes fingerprint searches through all available databases, referral to the New York Police Department (NYPD) Missing Persons Unit, full-body and dental radiography, an anthropological age analysis and radiographic review, odontological charting, upload to NamUs with searches against missing persons, and development of a DNA profile for upload to National CODIS. All unidentified decedents remain at NYC OCME until examination and documentation of the body is performed, a full identification investigation is conducted, and the case is reviewed by the IRC, which takes more than 1 month, but often longer depending on complexity. Once those actions are complete and the release is approved, unidentified decedents will receive a city-funded burial at Hart Island. Due to the above identification procedures (incorporated over the last decade), typically less than 25 out of approximately 9 000–10 000 individuals whose deaths are investigated by the NYC OCME remain unidentified more than a year after recovery, including those with tentative names that cannot be verified after extensive investigation. Despite the significant number of positive identifications (more than 900 identifications each month), some decedents remain unidentified for varying lengths of time, and their cases may later be investigated by the NYC OCME Long-Term Unidentified Persons Unit.

## The NYC OCME long-term UP investigation model

The NYC OCME is currently managing over 1 200 long-term UP cases in NamUs with most dating from the late 1980s, 1990s, and early 2000s, prior to the current advancements in forensic science, improvements in internal procedures, and development of the NamUs database. The NYC OCME Long-Term Unidentified Persons Unit performs investigations for these long-term UP cases, focusing on decedent identification. Performing investigations on cases that date back many decades can come with many complications, such as incomplete casefiles, poor documentation, lack of photos, inadequate UP DNA samples, the potential need to disinter UPs to collect additional biometrics, and minimal and/or incomplete data (including DNA) from missing persons (MP) cases for comparison. These obstacles require a more nuanced methodology and targeted use of resources when investigating leads, as well as the application of new forensic technologies. To do so effectively, it is important to review all available postmortem data and collect antemortem data to know what is available for comparison. Utilizing such an approach to long-term UP cases has resulted in the resolution of over 80 long-term UP cases ranging from 1969 to 2022. These procedures are described in more detail below with the use of NYC OCME case studies to illustrate how they are put into action.

### Postmortem data collection

The first step of any long-term UP investigation is the collection and review of all available postmortem data. The complete medical examiner casefile should be retrieved, as well as information that may be available such as the death certificate on file, archived photographs or radiographs, and reports from law enforcement, if available. The existing documentation should be reviewed with an understanding of available forensic technology and perspectives on social identity, both at the time the original report was generated and contemporary day. Each report provides the circumstances surrounding a UP’s death that could be used to generate identification hypotheses and, in turn, more effectively consider the best potential matches. The various types of postmortem data and how they can be carefully interpreted to form potential identification hypotheses is reviewed below.

### Scene findings

Scene findings speak to where, how, and in what condition an individual is found. A large-scale scene finding, such as recovery location, should be considered relative to its geographic and culturally significant location. For example, if an individual is recovered from an area notable for suicide (e.g. waterways), this finding can help hypothesize behaviour and inform MP comparisons. Someone who went missing after expressing suicidal ideation or who left home without any of their personal belongings may be a strong contextual match. Similarly, a decedent’s proximity to areas known for higher drug-use rates, undomiciled or transient populations (e.g. abandoned buildings or train lines), undocumented and/or migrant populations, and other culturally informative locations can also be used as potential leads for identification investigations.

A smaller scale scene finding is related to the individual within the scene and can convey additional information regarding decedent behaviour. For example, the body position may be consistent with an overdose, a terminal collapse, self-harm, or death that occurred during sleep. Although the identification investigation is not concerned with determining these outcomes, the body position and associated findings provide important context to consider when reviewing potential matches (e.g. did the MP have a known history of drug-use, suicidal ideation, or heart disease?).

### Personal effects

Clothing can be particularly useful as the brand, style, and even size or fit can speak to socioeconomic status, gender identity, cultural identity, social group identity, and even postmortem interval. Clothing can suggest a person’s interests or possible social group affiliations (e.g. employee, athlete, student, among many others). Logoed clothing can also have explicit affiliations, such as current or past employers or educational institutions, sports teams or political parties, or prior travel destinations. Clothing size can provide a basis for weight estimation or body size, even with skeletonized remains. Mismatched clothing and/or inappropriate size for the individual can suggest the clothing was obtained through donation. The cleanliness of clothing (disregarding postmortem damage) can imply varying personal hygiene standards or access. Clothing may also speak to a decedent’s mental state. If a decedent found outside is dressed inappropriately for the weather, it may suggest that they were intoxicated, experiencing delusion, or were not intending to be outside for any length of time. In cases of skeletonization, the date of manufacture for a particular item of clothing, footwear, or personal effect can assist in generating a possible postmortem interval.

Personal effects such as jewellery, key chains, handwritten notes, prescription bottles, cell phones, and items of religious significance are similarly valuable. Even seemingly insignificant items to an investigator may spark recognition in a friend or family member and therefore should be included in public postings. Especially in cases where a decedent is not visually recognizable because of decomposition or trauma, photographs of personal effects can be distributed in the hope they will be recognized.

Instead of focusing exclusively on the specific clothing and personal effects of the decedent in comparison to a potential MP, consider the overall impression of the items when evaluating identification leads. For example, several years ago the decomposed remains of an unidentified young male were found in a marshy area in Queens. He was not carrying identification, was visually unrecognizable, and fingerprints were not possible. He was wearing a blue and yellow windbreaker, a red T-shirt, military-issue wool pants, long black socks with cartoon giraffes pulled up to his calves and red skateboarding shoes with mismatched shoelaces. A NamUs search revealed a potential match from another borough in NYC. The MP NamUs posting was minimal and only contained basic physical descriptors, last seen alive date and location, and general clothing description including a blue jacket and gray pants. However, an internet search conducted by OCME investigators revealed that the missing young man was an avid skateboarder who typically wore long socks pulled up with skateboarding style shoes. Investigators contacted NYPD to collect antemortem records for comparison, and the young man was positively identified *via* a direct DNA comparison.

### Autopsy findings

Autopsy findings provide details about an individual’s physical characteristics, medical history, and even behaviour. This includes physical descriptors such as height, weight, eye colour, hair colour and length, facial hair, and skin tone. Medical findings, such as a history of disease, injuries, surgeries, and medical implants can provide informative leads. Distinctive features, such as scars, piercings, tattoos, amputations, or physical deformities can be clearly individualizing. However, the autopsy data provide far more than just the presence and description of these features. For example, various hairstyles and dyed hair colour can speak to membership within various social groups or habitual patterns of the unidentified decedent. The size, location, shape, and regularity of a scar can speak to histories of drug use, interpersonal violence, self-harm, or prior access (or lack thereof) to medical intervention. Tattoos are useful, not only for a direct comparison, but also as a physical display of the decedent’s interests or self-identity. Tattoos may have significance regarding region of origin, religion, military service, prior incarceration, gang affiliations, music preferences, birth sign, hobbies, gender identity, sexual preference, and possible sociocultural affiliations [2]. In addition, the quality and style of a tattoo can speak to an individual’s resources (e.g. a professional tattoo *versus* an amateur tattoo). In some cases, the tattoo may even contain the name, birthdate, or astrological sign of the decedent or a family member.

Autopsy findings of no apparent clinical significance may also be useful in the pursuit of an identification. These can include unusual physical characteristics, birthmarks, acne, nail pathologies, personal hygiene (e.g. presence of lice or scabies), and untreated conditions or infections, among many others. Each observation may provide clues regarding an individual’s access (or lack thereof) to financial, medical, and supporting resources, as well as their living conditions and possible mental status.

Similarly, the cause and manner of death for an individual may speak to specific antemortem experiences or state of mind around the time of death. All identifying information being equal, a UP witnessed to jump off a bridge is a stronger contextual lead for an MP who previously verbalized suicidal ideation and left all their personal belongings at home, over an MP with a significant medical history who left for work and never returned. Similarly, identification leads for a UP who died by homicide might suggest an MP who went missing under suspicious circumstances, engaged in risky behaviour or illegal activity, or experienced prior domestic violence.

### Toxicology findings

Toxicology findings are constructive for the medical examiner when determining the cause and manner of death, but in an identification context, they can also be used to help infer decedent behaviour. Toxicology reports can specify the presence of alcohol, illicit drugs, tobacco, synthetic hormones, antidepressants, antipsychotics, antianxiety medication, and medications to treat specific conditions. This can reflect medical histories and access to healthcare and prescriptions, as well as the possible mental state or behaviour around the time of death. Importantly, the absence of toxicology findings can be equally informative, indicating that an individual may not have been a habitual user of any of the above or may have been noncompliant with medications.

The toxicological results in consideration with other contextual information or postmortem findings, may be useful when assessing identification leads. One example of this is the recent identification of an unknown female who was recovered from the East River in 1993. This unknown female was markedly decomposed at the time she was recovered, a search of her fingerprints did not result in a hit, and DNA samples were not obtained in 1993. Toxicology results indicated Nortriptyline (an antidepressant) in her bloodstream. Her cause of death was drowning and the manner of death was undetermined at the time. There were no identification leads and she was buried at Hart Island in 1993. In 2022, the OCME FAU was contacted by a law enforcement agency regarding an MP for comparison. The MP had been depressed and known to be on antidepressants and had left all her belongings and a suicide note when she went missing. Although she was reported missing in an outside jurisdiction, she was familiar with NYC and would commute into the city regularly for work. Based on the consistent information, particularly the Nortriptyline and the history of depression, the medical examiner and MP records were reviewed for comparison and fortunately dental radiographs were located, allowing confirmation of the identification.

### Anthropology findings

An anthropological examination can assist in the generation of a biological profile (e.g. age, sex, population affinity, stature), as well as the assessment of antemortem trauma, identifying features for an unknown individual, and the estimation of postmortem interval. Anthropology has played an important role in decedent identifications at the NYC OCME since the 1980s, but it was not until the early-2000s when the process became more formalized, and analyses were conducted on individuals regardless of their state of preservation. Since then, the FAU has conducted an anthropological examination for all true unknown individuals, even when visually identifiable. The typical forensic anthropology examination of a true unknown will provide a scientific age estimate, as well as a detailed review of all full-body postmortem radiographs. If a decedent is decomposed or skeletonized to the extent that sex, population affinity, or stature estimations are necessary then those estimations are performed as well.

Anthropologists also analyze the skeletal remains and/or radiographs for any unique characteristics that could provide an identification lead, including anatomical variants, pathologies and untreated conditions, indicators of oral health, physiological stress indicators, prior injuries, and surgical intervention. It is not only the presence of these characteristics that is important, but also the suggestion of prior medical or dental interventions; a well-healed fracture may imply access to medical care, whereas a poorly set fracture may indicate lack of access. Additionally, if surgical hardware is observed on the postmortem radiograph, the forensic anthropologists will remove and assess the hardware for logos, lot numbers, or a serial number that can be used to provide a lead.

When reviewing long-term UP cases, an updated anthropological analysis should be performed on all true unknowns. In many instances, an anthropological analysis may have never been performed, regardless of the state of preservation. It is particularly important to reassess individuals who were decomposed, burned, or skeletonized, but it is worthwhile even if the decedent was visually identifiable. Confirmation of age, population affinity, sex, and postmortem interval estimates is a critical step to ensure the accuracy of postmortem details as errors may result in mistaken MP exclusions. Additionally, many anthropological methods have advanced considerably, meaning cases with prior anthropological examinations also require an updated review. It is ideal to do this through examination of the physical remains, but if that is impossible many estimates can be conducted using good quality photos and radiographs. A review of prior NYC OCME casefiles has produced many updated estimates that have contributed to long-term UP investigations. One example is of a young woman whose decomposed body was recovered from a vacant lot in 1991. At autopsy, her age was estimated as 20–40 years old. In 2022, someone called the OCME to conduct a comparison of this UP to a close friend who was 32 years old when she went missing in late 1990. Based on the circumstances and the original autopsy report, this appeared to be a promising lead and the remains were exhumed to obtain a DNA sample. Upon disinterment, an anthropological examination revealed that the individual had incomplete fusion of the medial clavicle epiphyses and incomplete formation of the third molar roots, indicating she was more likely 17–22 years of age. Based on this updated age-at-death information and lack of a DNA ranking in CODIS, the UP and MP were excluded as a match. A renewed identification investigation is now being conducted with the updated anthropological information and a UP DNA profile is searching at National CODIS.

### Dental findings

The dentition is useful in generating identification leads because it is highly visible and easily recognizable. Unfortunately, many of the long-term MP cases do not have available antemortem dental records for direct comparison. Therefore, aside from the description of the presence, location, and type of dental intervention common in postmortem dental charting, other findings such as visible gaps, rotated teeth, retained deciduous teeth, unusual or patterned dental wear, or discoloration may help generate links to an MP. These findings are also a useful point of comparison when a direct dental comparison is not possible. Similarly, because dental findings are easily visible and commonly recorded, they are great sources of data for efficiently determining exclusions.

Dental findings also provide information about resource availability and oral health behaviours. The presence of dental work can indicate access to dental resources (and potential dental records), whereas the presence of oral pathology with no intervention can indicate poor dental health with no access to such interventions. Importantly, because the permanent dentition forms during childhood and adolescence, dental findings can be useful in inferring changes in behaviour or socioeconomic status. For example, an individual may have several dental restorations, as well as numerous large caries and calculus build-up. The restorations indicate access to oral health care at some point in their life; however, the presence of active caries and calculus build-up suggest a lack of access to oral health care prior to death.

### Fingerprints

Between 2017 and early 2024, advances in fingerprint search capabilities have resulted in nearly 50 identification leads on long-term UP cases. Technological advances can produce “hits” (matches to an antemortem record) on fingerprints that were previously searched through the same database with negative results. Additionally, fingerprint databases continue to evolve as new sources of antemortem records are introduced. Thus, postmortem fingerprints should be periodically resubmitted to available databases wherever possible. It is strongly encouraged to upload all available postmortem biometric information to the NamUs UP posting (dental charting, radiographs, etc*.*), including the postmortem fingerprint card, even if they were previously searched as part of prior investigative efforts. If a tentative name or date of birth is available that should be provided to the FBI or Department of Homeland Security when submitting the fingerprints to allow for direct comparison to records with the same or similar demographic data. If possible military service, country of origin, or immigration information are known, it should also be provided so that it can be utilized for targeted records searches.

### Forensic biology

There have been impressive advances in DNA technology since the early 2000s that have made an impact on the identification of unknown individuals. Advances in STR, Y-STR, and mitochondrial deoxyribonucleic acid (mtDNA) technology, the broad development and implementation of CODIS, and genetic genealogy are some of the most impactful gains in DNA technology. Despite these advances, there are numerous constraints when working with long-term UP cases. Unfortunately, not all long-term UP and MP cases have available DNA searching at CODIS. The amount of time that has passed since UP burial or MP report filing, and resource constraints may require a targeted approach to determine which UP cases should be disinterred for additional testing and which MP families should be encouraged to submit family reference samples for comparison. A majority of the DNA identifications of NYC OCME long-term UP cases are resolved due to the targeted collection of MP and/or UP DNA samples for comparison, as opposed to blind CODIS matches. An additional barrier to be aware of is the hesitation of some families to provide reference samples for comparison. This discomfort can stem from numerous concerns, including but not limited to distrust of law enforcement or government agencies and a misunderstanding about how samples are used, further complicating the use of DNA in long-term UP cases. In these situations, it is crucial to work with families to address these fears, answering their questions, explaining investigative processes, and treating their concerns with respect.

Beyond CODIS uploads and one-to-one DNA comparisons, molecular analyses can provide additional lines of evidence for generating identification hypotheses for the long-term UP. mtDNA can be used to generate maps with the European DNA Profiling (EDNAP) Forensic mtDNA Population Database (EMPOP), which geographically plots haplotype hotspots. This helps visualize geographic regions with higher frequencies of the UP haplotype, which can provide additional predictions regarding population affinity and region of origin [3]. In addition to mtDNA, Y-STRs from unidentified biological males can be used to estimate haplotype frequency in the Y-Chromosome STR Haplotype Reference Database (YHRD). These searches provide frequency distributions of specific Y-STR haplotypes within global metapopulations and specific population groups, which can similarly help inform population affinity and region of origin [4]. While the EMPOP and YHRD region of origin findings can aid long-term UP investigations, it is important to emphasize that they should only be used in conjunction with anthropological, pathological, and contextual findings of population affinity.

Investigative Genetic Genealogy (IGG) has made a significant contribution to the identification of long-term UPs. Partnering with the NYPD, the borough District Attorney Offices, and non-governmental organizations, the NYC OCME has collaborated with external laboratories and genetic genealogists to resolve several of our most challenging long-term UP cases. In alignment with the interim Department of Justice policy and considering the resources required to conduct IGG, it is only utilized by NYC OCME after all investigative procedures have been completed and all identification leads have been exhausted.

### Other scientific findings

Other recent scientific technologies that have been utilized at the NYC OCME are stable isotope analysis and bomb curve dating. These are technologies that have been explored through collaboration with outside specialists [5, 6], and while they are still being evaluated, they show promise in narrowing down a possible time since death and a UP’s geographic origin.

### Collecting antemortem data for comparison

Gathering detailed, accurate, and comprehensive antemortem data for an MP is equally important when performing a comparison to a long-term UP and can often be complicated by numerous factors. Long-term UP investigations regularly involve comparisons to MPs from various timeframes, which can significantly impact the quantity and quality of antemortem data available.

Once a potential lead is generated, NYC OCME will gather and review antemortem information for the missing or presumed deceased person, often by reviewing public postings (e.g. social media, NamUs), working with law enforcement, or reviewing casefiles. This process is critical to determine what information specifically useful for identification purposes is readily available and what may still be needed. In many cases, the availability of certain information, such as dental or fingerprint records, is unknown and may require follow-up with law enforcement or families. Many times, the most efficient way to collect pertinent antemortem information is through interviews of those who knew the missing person. NYC OCME takes a collaborative and proactive approach to this task by working with law enforcement, as well as performing interviews directly with families or other informants. NYC OCME has specially trained staff who conduct interviews where targeted questions are asked to collect antemortem information solely for the purpose of performing morgue records searches and/or identification comparisons. Because of these specific goals, interview questions may differ from a general MP investigation and the level of detail collected may vary. See Table 1 for example questions that NYC OCME utilizes during an informant interview for a person who is missing and suspected to be deceased.

**Table 1:** Example questions utilized by New York City (NYC) Office of Chief Medical Examiner during an informant interview.

Topic	Question
General information	• What is their legal name? Do they use any nicknames? • What is their date of birth (DOB)? (Or age if DOB is unknown) • What is their sex? (considering both sex assigned at birth and gender presentation, if applicable) • What is their race? What is their ancestry?
Circumstantial information	• When were they last known to be alive (seen or spoken to)? Where? By whom? – If the exact date is unknown, what was the approximate year and season? – When was the last time you saw them? • Where were they last known to be alive? • Is there reason to believe they may have been in this area (New York/NYC)? Were they known to frequent specific local areas? • Was a missing person’s report filed with the police? • Were there any indicators about their medical or mental state around the time they went missing that may be important to consider? – Did they ever talk about mental health issues, addiction, or depression? – Did they ever express any suicidal ideations or self-harm behaviour? – Were they going through a difficult period in their life?
Physical appearance	• What is their height? • What is their weight? (or body habitus if weight is unknown) • What is their natural hair colour? Did they tend to dye their hair? – What was their hair texture like? Did they typically wear their hair in a specific style? • What is their eye colour? • Did they have any scars, skin abnormalities, or birthmarks? • Did they have any tattoos? – Did the tattoos symbolize anything? Were they in a specific style? Were they professionally done? • Did they have any piercings or other body modifications? • Was there anything unique about their teeth? – Were their teeth straight or were any teeth crooked? Were there any gaps, chips, or missing teeth? Did they ever get a filling? • Are there specific clothing or personal items they were last known to be wearing or have in their possession? • Did they have a certain type or style of clothing or jewellery that they habitually wore? How did their clothing typically fit? • Is there anything else distinct or recognizable about them?
Medical and social history	• Did they have any known medical or surgical history? • Did they take any medications? • Did they ever have any significant injuries or bodily trauma? If so, were they treated or go to a doctor for it? • Did they use tobacco? • Did they drink alcohol or use drugs? If so, is there a type they utilize most?
Antemortem records^a^	• Do you have any (recent) photographs of them that you can share? • Were they ever hospitalized or known to have X-ray/imaging taken? • Do you know if they have been to a dentist? If so, do you know the dentist information/location? • Were they known to have fingerprints on file for employment, immigration, arrests, etc*.*? – Did they ever serve in the military? • Are there biological relatives who would be willing to submit DNA, if needed? – What is their relationship? Are they maternally or paternally related?

a These questions may or may not be asked depending on the availability of postmortem information or based on necessity for identification purposes.

Similar to how holistic postmortem data analysis must consider both the individual details and the overall context of the case, antemortem data collection should assess specific data points as well as an overall impression of the MP’s circumstances and characteristics. NYC OCME will use the questions in Table 1 as a framework, but interviewers are trained to help elicit this type of general information if more detailed information is not known or available. For example, when asking for information regarding height, the interviewer may ask the informant to consider their own height and where the missing person may have stood in relation to them. Similarly, asking for a description of physicality such as build (e.g. slim, athletic, heavy-set) can be a useful substitute or complement to a weight estimate. Race and population affinity should be documented, but with the understanding that this is a fluid category that may not be accurately assessed in an unidentified decedent due to a variety of reasons including individual interpretation and postmortem changes. It may be difficult for an informant to provide an exact description of the clothing items an individual was wearing when they went missing, especially after a long time; therefore, it can be equally valuable to know the style or type of clothing they wore (e.g. baggy or fitted clothing, a specific colour palette, athletic/leisure wear). If the exact imagery of a tattoo cannot be described, the interviewer will ask about the style, themes, or possible significance. If the specific locations of the tattoos cannot be provided, documenting the general area can be useful, keeping in mind that it is not uncommon for someone to confuse which side of the body it appears on. When collecting information about medical history, interviewers inquire about any conditions, procedures, injuries that cause physical changes to the body (e.g. surgical scars, medical implants, oedema of the limbs, amputations), and any anomalies that could be a unique identifier.

Several techniques are utilized to help maximize the effectiveness of the interview. Interviewers will frequently provide examples or scenarios to help guide the informant in remembering specific details about the MP. Interviewers ask focused questions to help direct the interview, but will also allow the informant to provide information they feel useful or that has not been discussed by asking occasional questions, such as “is there anything else that may help us search our records?” Wherever possible, interviewers have already thoroughly reviewed applicable UP casefiles and are knowledgeable of available postmortem data. This will enable the interviewer to efficiently ask appropriate questions, collect detailed antemortem information, identify points of discrepancy or significance, and determine the general likelihood of a potential identification match.

In addition to reviewing available MP information and using comprehensive interviewing techniques, the investigation often requires searching additional resources and databases to fill in gaps that are necessary for a comparison. The amount of information readily available through general internet searches, social media, and sites such as NamUs is an invaluable resource. The NYC OCME investigative strategies also consider resources that were applicable and relevant at the time the person went missing. This may include census data, newspaper articles featuring MP postings, and National Archives. The ability to collect antemortem data suitable for comparison or morgue record searches relies on information sharing, a creative approach, and utilizing a combination of techniques and resources.

## Conclusion: a case study in holistic investigation

A lengthy investigation into the identity of an unknown female demonstrates not only the complexities that can arise, but also the importance of a holistic approach to identifications investigations. The partially skeletonized remains of an unknown female washed up on a beach in NYC in the mid-2010s. She exhibited several prominent and unusual professional tattoos, as well as a tattoo of an Eastern Orthodox cross on one finger. Her belly button was pierced and she had breast implants. The decedent was clad in a T-shirt and cropped leggings, both of which were faded and of indeterminate colour. An autopsy was performed and the cause and manner of death were both undetermined. An anthropological assessment estimated her age to be between 23 and 33; however, the population affinity was indeterminate. Fingerprints were captured using casting material that was dusted, photographed, and submitted to state, local, and federal databases, but produced no matches. The breast implant manufacturer was contacted, but the number on the implant was a lot number (i.e. not a unique identifier) and their records did not include a full list of patients who received that implant. Online searches (using Google, NamUs, and social media sites) were conducted for the individuals registered with that lot number to determine if any individuals aged 23 to 33 appeared to be missing or could be confirmed as alive based on activity after the date of death. Some individuals were ruled out, but no MP leads were generated from these searches. STR and mtDNA profiles were developed and submitted to CODIS. EMPOP (mtDNA) suggested a geographic origin most closely associated with Eastern Europe. Finally, an FBI Tattoo and Graffiti Team search was conducted; however, no additional information was located. Despite the abundance of seemingly informative postmortem data, the lack of comparable antemortem data hindered identification efforts.

After the initial identification investigation was complete, an investigator at the NYC OCME noted a newly created NamUs posting for an NYC missing woman in her late 20s, with a similar height, weight, and hair colour as the decedent, and a name suggestive of Eastern European heritage. Clothing was listed as pants and a shirt, described only by colour. The NamUs profile did not mention any tattoos, piercings, or breast implants and the woman’s publicly available Facebook page also did not reveal any photos of tattoos, although at one point she had “liked” the profile of an NYC plastic surgery clinic, suggesting a potential interest in body modification. NYPD was contacted for additional information regarding the MP. Further investigation revealed that her grandmother had filed the MP report, but was not aware of any tattoos, piercings, or breast implants. However, when investigators later spoke with another family member, they were able to verify the specific tattoos, piercings, and cosmetic surgery. Ultimately, an antemortem lateral cranium radiograph was located and used to confirm the identification. The remains were disinterred from Hart Island and returned to the family for private funeral arrangements.

This case illustrates the circuitous nature of how identification leads might be generated, particularly when the most logical first steps (interrogating surgical implant information, running fingerprints, investigating distinctive tattoos, submitting DNA to CODIS) fail to produce any meaningful leads. Ultimately, the connection between the MP and the UP was made by linking the Eastern-European name of the MP posted to NamUs to the UP’s hypothesized heritage as suggested by the EMPOP results and the Eastern Orthodox cross tattoo. This compelled the OCME to reach out to the NYPD for further information from the family. This speaks to the importance of a holistic investigation that considers all details known about an MP or UP and assessing all possible data for potential consistencies. Part of this process is accounting for who files an MP report and the different information they might know about the individual. As seen with this case, a grandmother might report different information than a sibling, friend, or partner.

Identifications investigations always require a nuanced approach, but long-term UP cases in particular demand a rigorous analysis of all available postmortem and antemortem data sources. This involves not only re-examining older casefiles and re-analyzing remains with newer techniques, but also re-assessing and re-formulating identification hypotheses, and applying a creative and flexible framework to identification investigations. This model has had notable success at the NYC OCME, resulting in over 80 long-term UP identifications confirmed between 2017 and early 2024 that date as far back as 1969. While every jurisdiction presents its own unique challenges, sharing this approach may be beneficial for practitioners in other jurisdictions.
